# Clinical Burden of People with Symptomatic and Exacerbating COPD While on Triple Inhaled Therapy †

**DOI:** 10.3390/jcm14186488

**Published:** 2025-09-15

**Authors:** Anthony De Soyza, Clementine Nordon, Emily Coak, Tia Pennant, Hana Mullerova, Malin Fageras, João Andre Alves, Wim Janssens

**Affiliations:** 1 Population and Health Sciences Institute, Faculty of Medical Sciences, Newcastle University, Newcastle NE2 4HH, UK; 2 Global Medical Evidence, AstraZeneca, Cambridge CB2 0AA, UK; 3 Adelphi Real World, Bollington SK10 5JB, UK; 4 Global Medical Affairs, AstraZeneca, 43183 Gothenburg, Sweden; 5 Department of Respiratory Diseases, University Hospital Leuven, 3000 Leuven, Belgium

**Keywords:** chronic obstructive pulmonary disease, exacerbations, triple inhaled therapy, disease burden

## Abstract

**Background/Objectives**: The burden of chronic obstructive pulmonary disease (COPD) in people who experience frequent moderate/severe exacerbations and frequent productive cough (FPC) while receiving triple inhaled therapy (TT) is poorly described. We aimed to examine clinical characteristics and patient-centric burden of this population. **Methods**: Data were drawn from the Adelphi COPD Disease-Specific Programme (DSP)™, a multinational cross-sectional survey on COPD (July 2022–January 2023). Patient characteristics and physician perceptions were described for the overall COPD cohort and subsets of those who were current/former **S**mokers, with ≥2 moderate or ≥1 severe **E**xacerbation(s) in the prior year while receiving **T**T and with FPC (SET+FPC) or without FPC (SET w/o FPC). **Results**: Of the overall cohort (N = 4372), 9.1% (*n* = 399) patients qualified for inclusion in the SET+FPC cohort; the mean (SD) age was 64.7 (10.9) and 69.1 (9.2) years, respectively. For the overall and SET+FPC cohorts, the mean (SD) number of exacerbations (prior year) was 1.1 (1.4) and 2.5 (1.4), respectively; median post-bronchodilator forced expiratory volume in 1 s (FEV1% predicted) was 68.0% and 49.0%, respectively, and physician-reported dyspnea grade (based on the modified Medical Research Council Dyspnea Scale) was ≥2 in 36.2% and 78.2%, respectively. Physicians were satisfied with COPD control in 76.1% of the overall and 39.6% of the SET+FPC cohorts. **Conclusions**: People with COPD who experience frequent moderate/severe exacerbations and FPC while receiving TT have substantial clinical and patient-centric burden and extensive unmet medical needs, suggesting inadequate disease control with current standard-of-care therapies.

## 1. Introduction

Chronic obstructive pulmonary disease (COPD) is the most prevalent noncommunicable respiratory disease and the third leading cause of death worldwide [1]. It is a heterogeneous lung condition characterized by chronic respiratory symptoms (dyspnea, cough, expectoration) due to persistent abnormalities of the bronchi (bronchitis), bronchioles (bronchiolitis), and/or alveoli (emphysema), which often result in progressive airflow limitation [2]. Exacerbations are also common, and over half of the people with COPD have been reported to experience exacerbations within a period of 5 years of diagnosis [3,4]. Airway inflammation and epithelial dysfunction are consistent and important features of COPD and are implicated in the pathogenesis and progression of the disease [5]. Chronic bronchitis is also prevalent in COPD, affecting 14% to 74% patients, and is associated with poor clinical outcomes [6,7,8]. Although chronic bronchitis is classically defined as having cough with sputum production for at least 3 months per year for 2 consecutive years [9], it is not always practical to capture this diagnosis in routine clinical practice.

Current COPD maintenance medication includes bronchodilation with inhaled long-acting β2-agonists and/or long-acting muscarinic antagonists. Inhaled corticosteroids are recommended treatment for people with recurrent exacerbations [9]. Beyond inhalers, oral add-on therapies (e.g., long-term macrolides, oral corticosteroids, phosphodiesterase-4 inhibitors, or mucolytics) may be considered; however, these have relatively poor risk–benefit profiles [9]. Given limited therapeutic options, it is estimated that up to 50% of individuals living with COPD and receiving triple inhaled therapy continue to experience exacerbations and remain symptomatic [10,11].

COPD is driven by multifaceted pathobiological processes involving goblet cell hyperplasia and mucous hypersecretion and multiple inflammatory pathways, including Type-1 and Type-3 (neutrophilic) inflammation, present in the majority of people with COPD, and Type-2 inflammation, present in 20% to 40% of people with COPD [12]. The pathophysiology of COPD likely varies between individuals, warranting the need for new treatments targeting the various pathobiological pathways in the airways; these are currently in development or have been recently approved and may provide disease-modifying opportunities for COPD [13,14]. Randomized controlled trials (RCTs) evaluating biologics in COPD target people who are symptomatic with a history of exacerbations while on (dual or) triple inhaled therapy. Trials of biologics in COPD targeting Type-2 inflammation include also high blood eosinophil count (BEC) as an eligibility criterion [15,16]. Notably, these RCTs often exclude clinically significant cardiovascular conditions or an asthma diagnosis, both of which are prevalent in people with COPD [13,14,15,16]. Little is known about the real-world characteristics of people who remain symptomatic and with exacerbations while on triple inhaled therapy and their disease burden beyond the frequency and severity of exacerbations. Smoking status and the presence of frequent productive cough (FPC) may also modify the real-world effectiveness of future biologics [17], warranting a more detailed understanding of the relationship of these factors with patient characteristics.

The main objective of this study was to describe people with COPD who had a history of smoking and a prescription for triple inhaled therapy and who experienced ≥2 moderate or ≥1 severe exacerbation in the past 12 months and FPC in the past 4 weeks (SET+FPC) and to contextualize their disease burden within the overall COPD population and, more specifically, with those not experiencing symptoms of FPC. Further, we have assessed the impact of smoking status within the primary SET+FPC population. The analysis captures physicians’ clinical impression correlated with patients’ symptoms, reflecting physicians’ typical assessment of COPD patients in real-world clinical practice.

## 2. Materials and Methods

### 2.1. Data Source

Data were drawn from the Adelphi Real World COPD Disease Specific Programme (DSP)™, a cross-sectional linked physician- and patient-reported survey, with retrospective data collection, of patients with COPD from France, Italy, Germany, Spain, the UK, China, and the US between July 2022 and January 2023. Adelphi Real World DSPs are set up independently of any specific study and are not designed to address any prespecified research questions or hypotheses. The methodology of the DSPs has been validated and used in previously published studies [18,19,20,21].

### 2.2. Source and Study Populations

#### 2.2.1. Overall Cohort

Primary care physicians or pulmonologists/respiratory specialists managing ≥ 3 COPD patients per month completed patient record forms for 5 to 8 consecutive outpatients who had a physician-confirmed COPD diagnosis; patients were excluded if they were aged < 18 years and involved in a clinical trial at the time of data collection. Participating patients were subsequently invited to complete a voluntary patient-reported survey. Each physician also retrospectively completed 1 to 2 additional patient record forms for patients receiving fixed triple therapy (“oversample”); patient-reported data were not captured for these patients (Supplementary Table S1).

#### 2.2.2. Study Populations

From the overall cohort, patients meeting key inclusion criteria used in RCTs for biologics were identified and classified as the “SET” subcohort (current/former **S**mokers, with ≥2 moderate or ≥1 severe **E**xacerbation in the prior year while receiving **T**riple inhaled therapy [22,23]. Patients in the SET subcohort had (1) a history of smoking (current or former smokers); (2) ≥2 moderate or ≥1 severe exacerbation in the 12 months preceding the survey date; and (3) a prescription for triple inhaled therapy at the date of survey. Patients in the SET subcohort were further categorized by presence of physician-reported frequent productive cough (FPC)/sputum in the 4 weeks preceding the survey date (yes or no); those with FPC were included in the “SET+FPC” subcohort, and those without FPC were included in the “SET w/o FPC” subcohort. The definition of FPC/sputum in the 4 weeks prior to survey date was chosen to highlight the burden of patients with COPD suffering from chronic cough/sputum. To avoid potential exclusion of patients who may not fulfil the strict definition for chronic bronchitis [24,25], FPC/sputum, based on physician assessment, was selected as a reasonable surrogate to chronic bronchitis in routine clinical practice. Patients in the SET+FPC subcohort were stratified also by smoking status (current or former smokers). The primary cohort of interest was the SET+FPC subcohort. Further context was provided by comparing the SET+FPC versus SET w/o FPC subcohorts and SET+FPC current versus SET+FPC former smoker subcohorts. The overall population was used to provide background burden in all COPD to better contextualize the burden experienced in these subcohorts.

### 2.3. Ethics

Using a checkbox, patients provided informed consent to take part in the survey. Data were collected (via assignment of a code) so that patients and physicians could not be identified directly; data were also pseudo-anonymized. Upon receipt by Adelphi Real World, data were pseudo-anonymized again to mitigate back tracing to the individual. Data were aggregated before sharing with the subscriber and/or for publication.

### 2.4. Measures

Physician-reported data included sociodemographics, cardiovascular conditions, asthma diagnosis, oxygen therapy use (ambulatory, long-term, or none), and COPD-related characteristics (most recent BEC and lung function, recent exacerbation patterns and severity, and impact of COPD daily symptoms). Lung function was reported using post-bronchodilator (post-BD) forced expiratory volume in 1 s (FEV_1_) as a percentage of the predicted value (FEV_1_% predicted). The degree of breathlessness was assessed by physicians based on the modified Medical Research Council (mMRC) Dyspnea Scale. Grades range from 0 to 4 (grade 0, least severe; grade 4, most severe); a dyspnea grade of ≥2 was used as a threshold for separating patients with “less breathlessness” from “more breathlessness” per the Global Initiative for Chronic Obstructive Lung Disease (GOLD) guidelines [9,26].

Additionally, physicians reported their satisfaction with the level of current COPD control for patients and whether they believed the best possible control was achieved. They provided their perception of COPD “severity” for patients at initiation of current treatment (i.e., triple inhaled therapy) and at the time of survey.

Patient-reported data included the COPD Assessment Test (CAT; scores range from 0 to 40, with higher scores reflecting greater symptom severity) and the European Quality of Life Visual Analogue Scale (EQ-VAS; scores range from 0 to 100, reflecting the worst and best imaginable health state, respectively).

Physician-reported data forms and patient-reported forms were linked via a unique, pseudo-anonymized number. All information was recorded on the survey completion date from available medical history or specified recall period for physicians and patients. No follow-up information was collected.

### 2.5. Data Analysis

Data were described using means (SD), medians (quartile [Q]1, Q3), or frequencies and percentages, as appropriate. Although this analysis was descriptive and cross-sectional in nature, an exploratory bivariate analysis was used to compare outcome variables between cohorts; *t*-tests were used for numeric variables, Fisher’s Exact tests were used for categorical variables (Chi-squared tests were used when Fisher’s Exact could not be calculated), and Mann–Whitney U (non-parametric) tests were used for ordered categorical variables. Comparisons were made between (1) the SET+FPC vs. SET w/o FPC cohorts and (2) the SET+FPC current smoker vs. SET+FPC former smoker cohorts. A significance level of α = 0.05 was used, and all tests performed were two-sided in nature.

Missing data were reported and not imputed. All statistical analyses were performed in Stata 18 (StataCorp. 2023. Stata Statistical Software: Release 18. College Station, TX, USA: StataCorp LLC). All descriptive analyses were conducted using IBM^®^ SPSS^®^ Data Collection Survey Reporter version 7.5.

## 3. Results

**Figure 1:**
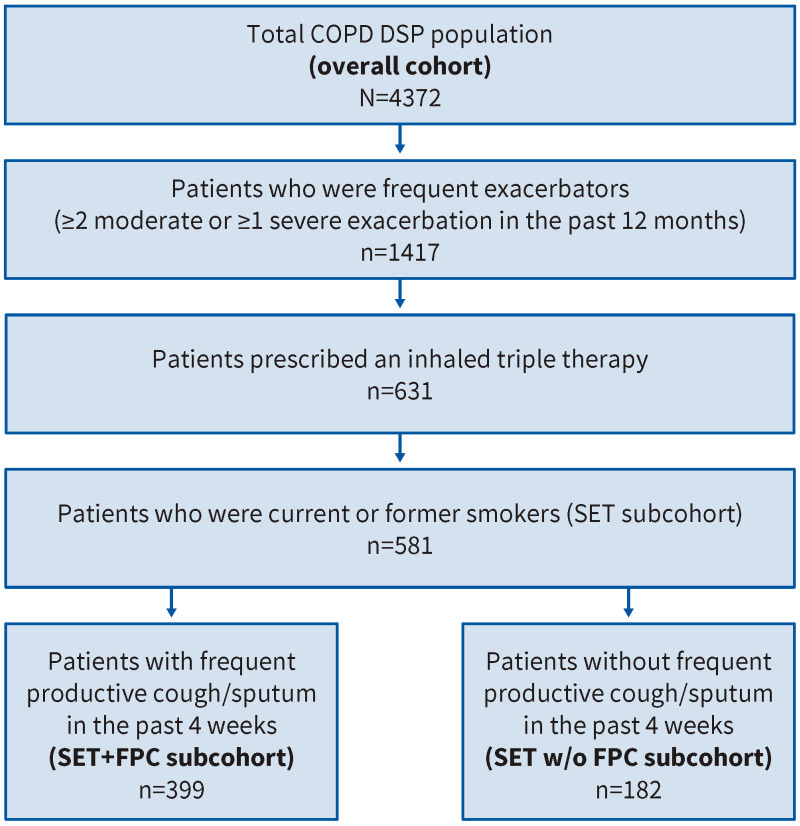
Patient flow chart.

A total of 837 physicians participated in the DSP (313 primary care physicians and 524 respiratory specialists) and provided information for 4372 patients (overall cohort). From the overall cohort, 581 patients (13.3%) formed the SET subcohort; 399 patients (9.1%) also had FPC (SET+FPC subcohort), and 182 patients (4.2%) did not (SET w/o FPC subcohort) (Figure 1).

### 3.1. Sociodemographic and Medical Conditions

Sociodemographics, comorbid cardiovascular conditions, and COPD-related characteristics of the overall cohort and the SET+FPC subcohort are summarized in Table 1 and Supplementary Table S2. Patients in the overall cohort had a mean (SD) age of 64.7 (10.9) years with a male-to-female ratio of 2.1 to 1. Patients in the SET+FPC subcohort had a mean (SD) age of 69.1 (9.2) years and a male-to-female ratio of 2.6 to 1. In both the overall and SET+FPC subcohorts, former smokers (57.4% and 62.4%, respectively) were more prevalent than current smokers (30.7% and 37.6%, respectively). Hypertension was the most common cardiovascular condition in both the overall (49.1%) and the SET+FPC (64.2%) cohorts. Oxygen therapy was received by 16.5% and 35.1% of the overall and SET+FPC cohorts, respectively. Information on BEC was available for only 13.2% (*n* = 579) of the overall cohort and 14.3% (*n* = 57) of the SET+FPC subcohort and revealed that in both groups, nearly one-third of patients had BEC ≥ 300 cells/μL (27.1% and 29.8%, respectively). Median post-BD FEV_1_ (% predicted) was 68.0% in the overall cohort and 49.0% in the SET+FPC subcohort, which reflected mild, moderate, severe, and very severe airway obstruction in 24.7%, 55.7%, 15.4%, and 4.2% of patients, respectively, in the overall cohort and 7.0%, 39.0%, 44.4%, and 9.6%, respectively, in the SET+FPC subcohort. In the overall cohort, 9.6% of patients had ≥2 moderate exacerbations in the prior year (vs. 32.1% in the SET+FPC subcohort), and 22.8% had ≥1 severe exacerbation (vs. 67.9% in the SET+FPC subcohort).

**Figure 2:**
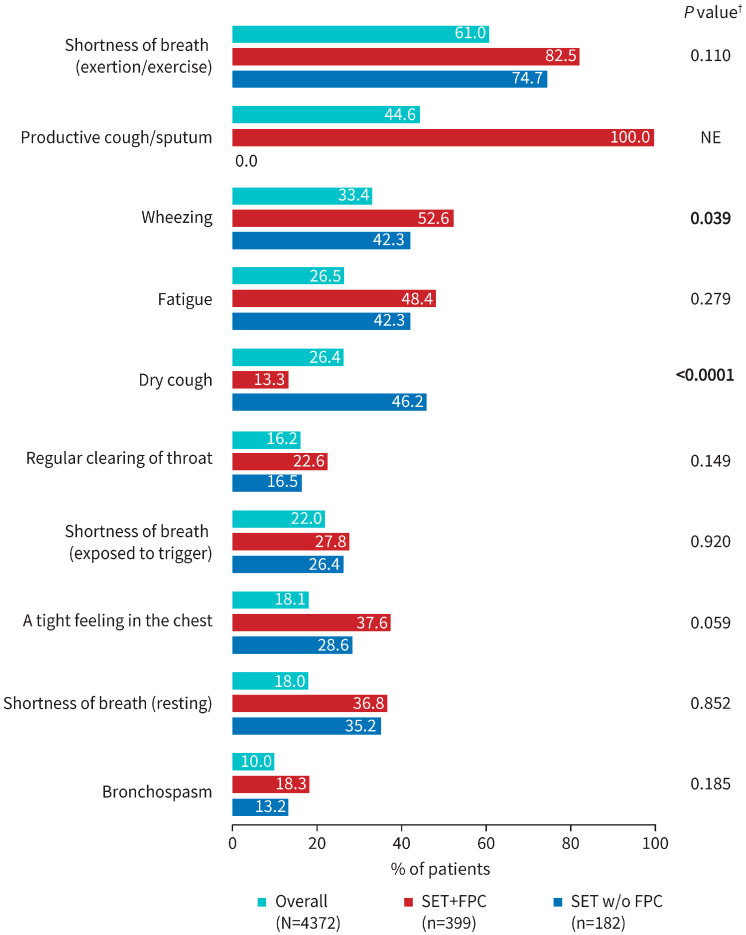
Respiratory symptoms frequently reported by physicians for the overall, SET+FPC (current/former Smokers, with ≥2 moderate or ≥1 severe Exacerbation in the prior year while receiving Triple inhaled therapy [SET] and frequent productive cough [FPC]), and SET w/o FPC cohorts. ^†^ Statistical analysis was conducted for the SET+FPC (primary cohort) and SET w/o FPC cohort using Fisher’s Exact test. No comparisons with the overall cohort were conducted as it includes the SET+FPC and SET w/o FPC cohorts and is only provided for context.

Dyspnea grade ≥ 2 was reported for 36.2% of patients in the overall cohort and 78.2% in the SET+FPC subcohort. Respiratory symptoms commonly reported by physicians are displayed in Figure 2 for the overall and SET+FPC cohorts. Shortness of breath, wheezing, and fatigue were the most frequently reported symptoms in both cohorts; FPC was present in 44.6% of overall cohort patients.

The SET+FPC subcohort categorized by smoking status is described in Supplementary Table S3. Current smokers were significantly younger on average than former smokers (mean [SD] age of 65.7 [8.6] and 71.2 [9.0] years, respectively; *p* < 0.0001) and less frequently male (65.3% and 76.3%, respectively; *p* = 0.021); median post-BD FEV_1_ (% predicted) values were comparable (50.0% and 48.0%, respectively), and 71.3% of current smokers and 65.9% of former smokers had ≥1 severe exacerbation in the prior year. Higher proportions of former smokers had congestive heart failure (12.4% vs. 4.0%; *p* = 0.004) and cardiac arrhythmias (23.3% vs. 14.0%; *p* = 0.027) compared with current smokers. Among respiratory symptoms, dry cough was present in 12.0% and 14.1% of current and former smokers, respectively (Supplementary Figure S1).

For 182 patients in the SET w/o FPC subcohort, the mean (SD) age was 67.2 (9.3) years; 68.1% were male and 39.6% were current smokers (Table 1). Median post-BD FEV_1_ (% predicted) was similar to patients in the SET+FPC subcohort (49.5% vs. 49.0); however, significantly fewer patients in the SET w/o FPC subcohort (vs the SET+FPC subcohort) had dyspnea grade ≥ 2 (63.7% vs. 78.2%; *p* = 0.0004) and a higher proportion of patients in the SET w/o FPC subcohort had ≥1 severe exacerbation in the year prior to the survey date (79.1% vs. 67.9%; *p* = 0.010). Dry cough was also more prevalent in the SET w/o FPC subcohort vs. the SET+FPC subcohort (46.2% vs. 13.3%; *p* < 0.0001) (Figure 2).

### 3.2. Physicians’ Satisfaction with COPD Control

**Figure 3:**
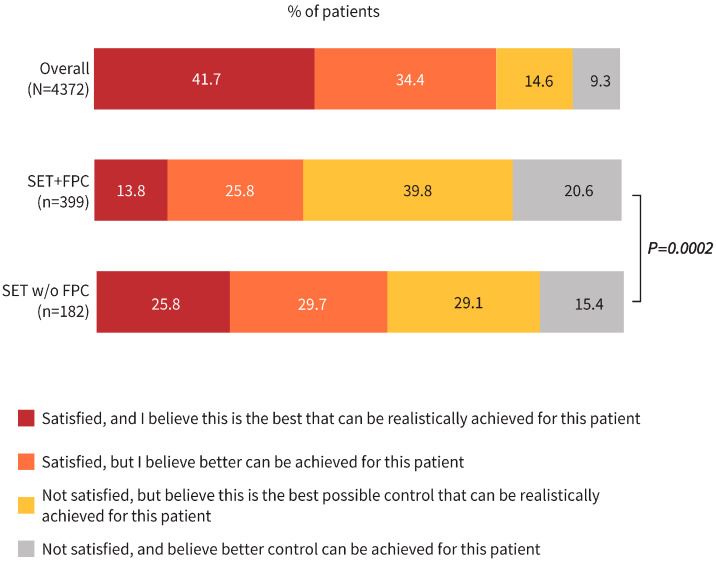
Physician satisfaction with COPD control for the overall, SET+FPC (current/former Smokers, with ≥2 moderate or ≥1 severe Exacerbation in the prior year while receiving Triple inhaled therapy [SET] and frequent productive cough [FPC]), and SET w/o FPC cohorts. Notes: Statistical analysis was conducted for the SET+FPC (primary cohort) and SET w/o FPC cohort using the Mann–Whitney U test. No comparisons with the overall cohort were conducted as it includes the SET+FPC and SET w/o FPC cohorts and is only provided for context.

Physicians’ satisfaction with the level of COPD control and their perception on whether better control could be achieved are reported for all patient groups in Figure 3 and Figure S2. In the overall cohort, physicians were satisfied with the level of COPD control for 76.1% of patients (Figure 3). In the SET+FPC subcohort, physicians were satisfied with the level of COPD control for 39.6% of patients, including 13.8% for whom they thought that the current control level reflected the “best that could be realistically achieved.” In 20.6% of the SET+FPC subcohort (25.3% of current smokers and 17.7% of former smokers), physicians were not satisfied with COPD control and thought that “better control could be achieved” (Figure 3 and Figure S2). In the SET w/o FPC subcohort, physicians were satisfied with COPD control for 55.5% of patients, including 25.8% for whom they thought that the current control level reflected the “best that could be realistically achieved” (Figure 3). Physician responses for the SET+FPC subcohort significantly differed from responses for the SET w/o FPC subcohort (*p* = 0.0002), while no significant differences were observed between current and former smokers in the SET+FPC subcohort (*p* = 0.175) (Figure 3 and Figure S2).

### 3.3. Physician-Reported Severity of Patient COPD Symptoms

In the overall cohort, the proportion of patients considered by their physicians as having “severe” COPD was comparable at the time of current COPD treatment initiation and on the survey completion date (17.4% and 15.2%, respectively; Supplementary Figure S3). The proportion of patients considered as having “moderate” COPD was 53.1% at current treatment initiation and 45.7% at survey completion. In the SET+FPC subcohort, the proportion of patients considered as having “severe” COPD was 50.1% at current COPD treatment initiation and 50.6% at survey completion. Results for patients in the SET+FPC subcohort stratified by smoking status and SET w/o FPC subcohort are also shown in Supplementary Figure S3.

### 3.4. Patient-Reported Outcome Measures

Patient-reported CAT and EQ-VAS scores for the overall, SET+FPC, and SET w/o FPC cohorts are reported in Table 2. Mean (SD) CAT scores were 17.9 (8.6) for the overall cohort and 25.0 (7.6) for the SET+FPC subcohort; mean (SD) EQ-VAS scores were 67.2 (17.7) and 52.9 (17.6), respectively. CAT and EQ-VAS scores for the SET+FPC subcohort stratified by smoking status are reported in Supplementary Table S4.

## 4. Discussion

In this multinational cross-sectional survey of 4372 individuals with COPD, almost 1 in 10 patients had a history of smoking and continued to experience a substantial burden from COPD, with frequent moderate or severe exacerbations and FPC while on triple inhaled therapy [27,28]. More than two-thirds of patients in this subcohort had at least 1 severe exacerbation in the past year, and more than three-quarters had clinically significant dyspnea. Daily symptoms beyond dyspnea were frequent, with wheezing and fatigue experienced by approximately half of patients; patient-reported outcomes also revealed poor health-related quality of life (HRQOL) based on elevated CAT and low EQ-VAS scores. Despite having high symptom and exacerbation burden, physicians were satisfied with disease control for 1 in 4 of this patient population.

In this study, patients who had not stopped smoking (38% of the SET+FPC subcohort) were significantly younger and had lower proportions with congestive heart failure and cardiac arrhythmia than former smokers. However, physician-reported levels of dyspnea, lung function, and exacerbation frequency and patient-reported health state scores were similar between the groups. Smoking cessation is a key component of COPD management and poses a challenge for patients [9,29]. Although not significant, we found a higher proportion of physician-reported dissatisfaction with COPD control, believing that better control could be achieved, for patients who had not yet stopped smoking (25%) than those who had stopped smoking (18%). These results collectively highlight the burden of disease and the imperative to improve overall management and therapies for severe COPD patients, irrespective of smoking status.

Patients in the SET w/o FPC subcohort comprised 4% of the overall cohort and were described to gain insight into the impact of FPC on COPD symptoms, physicians’ perception, and HRQOL. In contrast with previous reports, this study revealed that severe exacerbations were more prevalent among patients without FPC than with FPC (79% vs. 68%, respectively), while lung function was similar between the cohorts [25,30,31]. The proportion of patients with clinically significant dyspnea was significantly lower in the SET w/o FPC subcohort than in the SET+FPC subcohort, and the most frequent symptom for the SET w/o FPC subcohort was dry cough. While other diagnoses should be considered, given the exacerbation profile of the SET w/o FPC cohort while on triple inhaled therapy, it is likely that some patients in the SET w/o FPC subcohort may have had silent mucous plugs (i.e., mucous plugs identified on computed tomography scans without the symptom of FPC). A recent study showed that 36% of COPD patients without FPC had silent mucous plugs, and these patients had higher odds of severe exacerbation (vs. patients without mucous plugs) [32]. Interestingly, physician satisfaction with the level of COPD control was significantly higher in the SET w/o FPC subcohort compared with the SET+FPC subcohort, demonstrating the burden of FPC in patients with COPD and the need for better management of this symptom. Disease severity may be underestimated (and disease control overestimated) in patients with silent mucous plugs; however, this study did not capture the presence of silent mucous plugs.

Of the overall cohort, about 10% met the eligibility criteria for inclusion into the SET+FPC subcohort. Although this proportion seems low, due to the cross-sectional nature of the study, this proportion is likely to be underestimated, because people with COPD at some point over the course of their disease may exhibit the required symptoms to meet these criteria. Also, considering the global prevalence of COPD (about 392 million), 10% reflects a considerable number of people living with frequent/severe exacerbations and FPC while receiving triple inhaled therapy [33].

The impact of COPD on patient HRQOL has been previously studied, and total CAT scores observed for patients in the SET+FPC subcohort align with results for “very severe” COPD patients in a cross-sectional European study [34]. While many HRQOL studies have been conducted at the national level and in broad COPD cohorts, this study offers a global view of HRQOL in patients with COPD with insights into particular subgroups (patients with frequent/severe exacerbations with or without FPC and current/former smokers). Results show that while patients in the overall cohort reported lower EQ-VAS scores compared with population norms [35], scores were further reduced in the SET+FPC subcohort. Considered in conjunction with prior studies [34,36], these data herein highlight the ongoing disease burden and need for better therapies in COPD. Overall, these findings improve our understanding of the clinical burden in COPD patients who continue to experience symptoms while receiving standard of care (i.e., triple inhaled therapy) and of their physicians’ perceptions of COPD management. The unmet needs identified in this study may act as a call to action for practitioners, payers, and policymakers and help define the size, scope, and characteristics of patients who may require additional treatment, particularly with biologics that are expected to emerge and gain approval over the next 2 to 5 years [13].

Several limitations to the study may be noted. The Adelphi COPD DSP surveys were completed by general practitioners and pulmonologists to maximize the representativeness of patients; however, physician selection bias is possible as minimal exclusion criteria governed selection, and participation was influenced by willingness to complete the survey and practical considerations such as geographical location. Patient selection bias is also possible, as participating patients were identified based on physician judgement and perception rather than medical coding (e.g., diagnostic codes); however, we believe that this process is representative of physicians’ real-world classification of patients across many countries. To minimize this bias, physicians were asked to provide data on consecutively consulting patients who met the eligibility criteria. The DSP does not represent a truly random sample of patients, as the methodology states for 5 to 8 COPD patients who consecutively consult with the physician to be included, thus restricting the generalizability of the findings. Physicians and patients were requested to capture patient information retrospectively in the survey, which may introduce recall bias, a common limitation of surveys. However, the data for these analyses were collected at the time of each patient’s consultation and physicians were able to refer to patients’ medical records while completing the patient record forms, potentially minimizing this bias. Missing data were not imputed; therefore, the sample of patients included in each analysis could vary. The presence of FPC (in the past 4 weeks) was physician-reported and based on medical records, not objective measures; however, this is likely to reflect real-world diagnostic practice. It is likely that some patients with more severe COPD may have had coexistent bronchiectasis as well as other codiagnoses, which could have contributed to the FPC noted; however, the presence of coexistent bronchiectasis was not assessed in this study. Future studies with additional computed tomography imaging are recommended to understand the impact of coexistent bronchiectasis and the presence of silent mucous plugs in COPD.

## 5. Conclusions

This study demonstrates the substantial clinical, societal, and healthcare burden experienced by a subset of patients experiencing exacerbations and FPC while receiving triple inhaled therapy. While around 10% of the COPD population qualified in this subcohort, this proportion might be higher in routine clinical practice. Importantly, physicians wanted better control for more than 60% of these patients but felt this was the best currently attainable for most of them. The unmet needs identified in this study may help define the COPD patient population in need of additional treatment due to inadequate symptom management. Future research into improving COPD management will likely require both targeted therapies against airway inflammation and epithelial dysfunction coupled with renewed attention to smoking cessation.

## Figures and Tables

**Table 1:** Sociodemographics, cardiovascular conditions, COPD-related characteristics, and current treatment duration among patients in the overall, SET+FPC (current/former Smokers, with ≥2 moderate or ≥1 severe Exacerbation in the prior year while receiving Triple inhaled therapy [SET] and frequent productive cough [FPC]), and SET w/o FPC cohorts.

	Overall N = 4372	SET+FPC *n* = 399	SET w/o FPC *n* = 182	*p* Value *
Mean (SD) age, years	64.7 (10.9)	69.1 (9.2)	67.2 (9.3)	**0.019**
Male, *n* (%)	2965 (67.8)	288 (72.2)	124 (68.1)	0.375
Smoking status, *n* (%)				0.713
Current smoker	1343 (30.7)	150 (37.6)	72 (39.6)	
Former smoker	2510 (57.4)	249 (62.4)	110 (60.4)	
Never smoked	441 (10.1)	n/a ^†^	n/a ^†^	
Unknown	78 (1.8)	n/a ^†^	n/a ^†^	
Cardiovascular and metabolic diseases, *n* (%)				
Hypertension	2148 (49.1)	256 (64.2)	103 (56.6)	0.097
Elevated cholesterol/hyperlipidemia	987 (22.6)	114 (28.6)	45 (24.7)	0.367
Coronary artery disease	393 (9.0)	51 (12.8)	25 (13.7)	0.791
Cardiac arrhythmias	355 (8.1)	79 (19.8)	17 (9.3)	**0.002**
Congestive heart failure	206 (4.7)	37 (9.3)	16 (8.8)	1.000
Cerebrovascular disease	174 (4.0)	32 (8.0)	10 (5.5)	0.305
Angina pectoris	158 (3.6)	26 (6.5)	15 (8.2)	0.486
Myocardial infarction	148 (3.4)	25 (6.3)	14 (7.7)	0.592
Diabetes without chronic complication	601 (13.7)	78 (19.5)	33 (18.1)	0.734
Diabetes with chronic complication	143 (3.3)	24 (6.0)	5 (2.7)	0.104
Asthma codiagnosis, *n* (%)	123 (2.8)	13 (3.3)	5 (2.7)	1.000
Oxygen therapy use, *n* (%)				0.7703
Ambulatory	404 (9.2)	57 (14.3)	22 (12.1)	
Long-term	317 (7.3)	83 (20.8)	37 (20.3)	
None	3651 (83.5)	259 (64.9)	123 (67.6)	
Most recent BEC, cells/μL ^‡^				
Patients with missing data	*n* = 3793	*n* = 342	*n* = 148	
Patients included	*n* = 579	*n* = 57	*n* = 34	
Mean (SD)	337.7 (594.2)	277.5 (250.1)	306.2 (664.4)	0.770
Median (Q1, Q3)	184.0 (120.0, 300.0)	200.0 (124.0, 300.0)	193.5 (104.5, 254.5)	
<150, *n* (%)	184 (31.8)	16 (28.1)	13 (38.2)	0.242
150–299, *n* (%)	238 (41.1)	24 (42.1)	14 (41.2)	
≥300, *n* (%)	157 (27.1)	17 (29.8)	7 (20.6)	
Most recent post-BD FEV_1_ (% predicted) ^‡^				
Patients with missing data	*n* = 2535	*n* = 212	*n* = 96	
Patients included	*n* = 1837	*n* = 187	*n* = 86	
Mean (SD)	65.4 (17.9)	50.2 (16.5)	48.9 (15.7)	0.517
Median (Q1, Q3)	68.0 (55.0, 79.0)	49.0 (38.0, 62.0)	49.5 (38.0, 61.0)	
Degree of airflow obstruction (GOLD stage), *n* (%)				0.984
≥80% (GOLD 1 [mild])	454 (24.7)	13 (7.0)	2 (2.3)	
50–79% (GOLD 2 [moderate])	1024 (55.7)	73 (39.0)	41 (47.7)	
30–49% (GOLD 3 [severe])	282 (15.4)	83 (44.4)	33 (38.4)	
<30% (GOLD 4 [very severe])	77 (4.2)	18 (9.6)	10 (11.6)	
Pattern of exacerbations in the last 12 months				**0.010**
None or mild, *n* (%)	2281 (52.2)	n/a ^†^	n/a ^†^	
1 moderate, *n* (%)	674 (15.4)	n/a ^†^	n/a ^†^	
≥2 moderate (no severe), *n* (%)	421 (9.6)	128 (32.1)	38 (20.9)	
≥1 severe (±moderate), *n* (%)	996 (22.8)	271 (67.9)	144 (79.1)	
Mean (SD) number of exacerbations	1.1 (1.4)	2.5 (1.4)	2.3 (1.3)	0.073
Breathlessness (dyspnea scale), *n* (%) ^¶^				**0.004**
Grade 0: Only gets breathless after strenuous exercise	1181 (27.0)	14 (3.5)	11 (6.0)	
Grade 1: Gets breathless when hurrying on level ground or walking up a slight incline	1610 (36.8)	73 (18.3)	55 (30.2)	
Grade 2: On level ground, walks slower than people of the same age because of breathlessness or has to stop for breath when walking	959 (21.9)	142 (35.6)	58 (31.9)	
Grade 3: Stops for breath after walking a few minutes on level ground	464 (10.6)	134 (33.6)	35 (19.2)	
Grade 4: Is too breathless to leave the house or becomes breathless when getting dressed	158 (3.6)	36 (9.0)	23 (12.6)	
Patients with a breathlessness score grade ≥ 2	1581 (36.2)	312 (78.2)	116 (63.7)	**0.0004**
FPC in the last 4 weeks, *n* (%)	1948 (44.6)	399 (100.0) ^§^	n/a ^†^	
Prescription of triple inhaled therapy, *n* (%)	1205 (27.6)	399 (100.0) ^§^	182 (100.0) ^§^	
Duration of current treatment, weeks ^#^				
Mean (SD)	69.7 (112.3)	54.8 (72.1)	55.2 (62.2)	0.957
Median (Q1, Q3)	48.0 (12.0, 80.0)	36.0 (12.0, 54.0)	42.5 (17.8, 71.2)	

Notes: The SET+FPC and SET w/o FPC cohorts are subsets of the overall cohort and defined as patients with a history of smoking and ≥2 moderate or ≥1 severe exacerbation while on triple inhaled therapy, with or without FPC, respectively. * *p*-value comparison between the SET+FPC (primary cohort) and SET w/o FPC cohort; statistical analyses were conducted using T-tests, Fisher’s Exact tests, and Mann–Whitney U tests as appropriate (*p* < 0.05 indicated in bold). No comparisons with the overall cohort were conducted as it includes the SET+FPC and SET w/o FPC cohorts and is only provided for context. ^†^ None recorded for patients in the subcohort per inclusion criteria. ^‡^ For patients with available information. ^¶^ Based on the modified Medical Research Council dyspnea scale. ^§^ Recorded for all patients in the subcohort per inclusion criteria. ^#^ Based on physician-reported estimates of treatment duration. BD, bronchodilator; BEC, blood eosinophil count; COPD, chronic obstructive pulmonary disease; FEV_1_, forced expiratory volume in 1 s; FPC, frequent productive cough; GOLD, Global Initiative for Chronic Obstructive Lung Disease; Q, quartile; SET, current or former smokers with frequent/severe exacerbations while on triple inhaled therapy; w/o without.

**Table 2:** Patient-reported CAT and EQ-VAS scores for the overall, SET+FPC (current/former Smokers, with ≥2 moderate or ≥1 severe Exacerbation in the prior year while receiving Triple inhaled therapy [SET] and frequent productive cough [FPC]), and SET w/o FPC cohorts.

	Overall N = 4372	SET+FPC *n* = 399	SET w/o FPC *n* = 182	*p* Value *
CAT total score ^†^				
Patients with missing data	*n* = 2497	*n* = 271	*n* = 113	
Patients included	*n* = 1875	*n* = 128	*n* = 69	
Mean score (SD)	17.9 (8.6)	25.0 (7.6)	22.8 (8.8)	0.076
Median (Q1, Q3)	18.0 (11.0, 24.0)	26.0 (21.0, 30.0)	24.0 (16.0, 30.0)	
CAT cough domain score ^†,‡^				
Patients with missing data	*n* = 2535	*n* = 272	*n* = 115	
Patients included	*n* = 1837	*n* = 127	*n* = 67	
<2, *n* (%)	395 (21.5)	7 (5.5)	4 (6.0)	1.000
≥2, *n* (%)	1442 (78.5)	120 (94.5)	63 (94.0)	
CAT phlegm/sputum domain score ^†,¶^				
Patients with missing data	*n* = 2535	*n* = 272	*n* = 115	
Patients included	*n* = 1837	*n* = 127	*n* = 67	
<2, *n* (%)	524 (28.5)	13 (10.2)	14 (20.9)	0.051
≥2, *n* (%)	1313 (71.5)	114 (89.8)	53 (79.1)	
EQ-VAS ^†^				
Patients with missing data	*n* = 2510	*n* = 271	*n* = 113	
Patients included	*n* = 1862	*n* = 128	*n* = 69	
Mean (SD) score	67.2 (17.7)	52.9 (17.6)	57.6 (19.6)	0.084

Notes: The SET+FPC and SET w/o FPC cohorts are subsets of the overall cohort and defined as patients with a history of smoking and ≥2 moderate or ≥1 severe exacerbation while on triple inhaled therapy, with or without FPC, respectively. * *p*-value comparison between the SET+FPC (primary cohort) and SET w/o FPC cohort; statistical analyses were conducted using T-tests and Fisher’s Exact tests, as appropriate. No comparisons with the overall cohort were conducted as it includes the SET+FPC and SET w/o FPC cohorts and is only provided for context. ^†^ For patients with available information. ^‡^ The cough item on the CAT is scored on a scale of 0 (“I never cough”) to 5 (“I cough all the time”). ^¶^ The phlegm/sputum item on the CAT is scored on a scale of 0 (“I have no phlegm in my chest at all”) to 5 (“My chest is completely full of phlegm”). CAT, COPD Assessment Test; COPD, chronic obstructive pulmonary disease; EQ-VAS; European Quality of Life Visual Analogue Scale; FPC, frequent productive cough; Q, quartile; SET, current or former smokers with frequent/severe exacerbations while on triple inhaled therapy; w/o, without.

## Data Availability

The authors confirm that the data supporting the findings of this study are available within the article.

## Supplementary Materials

The following supporting information can be downloaded at https://www.mdpi.com/article/10.3390/jcm14186488/s1, Table S1: Physician and patient cohorts across countries; Table S2: Additional sociodemographics of patients in the overall, SET+FPC, and SET w/o FPC cohorts; Table S3: Sociodemographics, cardiovascular conditions, COPD-related characteristics, and current treatment duration among patients in the SET+FPC subcohort stratified by smoking status; Table S4: Patient-reported CAT and EQ-VAS scores for the SET+FPC subcohort stratified by smoking status; Figure S1: Respiratory symptoms frequently reported by physicians for patients in the SET+FPC subcohort stratified by smoking status; Figure S2: Physician satisfaction with COPD control for the SET+FPC subcohort stratified by smoking status; Figure S3: Physician-reported severity of COPD in patients, from time of current treatment initiation to time of survey for the overall, SET+FPC (overall and stratified by smoking status), and SET w/o FPC cohorts.

## Abbreviations

The following abbreviations are used in this manuscript:

BD	Bronchodilator
BEC	Blood eosinophil count
CAT	COPD Assessment Test
COPD	Chronic obstructive pulmonary disease
DSP	Disease Specific Programme
EQ-VAS	European Quality of Life Visual Analogue Scale
FEV1	Forced expiratory volume in 1 s
FPC	Frequent productive cough
GOLD	Global Initiative for Chronic Obstructive Lung Disease
HRQOL	Health-related quality of life
mMRC	Modified Medical Research Council
NE	Not evaluable
Q	Quartile
RCT	Randomized controlled trials
SET	Current or former smokers with frequent/severe exacerbations while on triple inhaled therapy
