# Supplementary material for: Clinical Burden of People with Symptomatic and Exacerbating COPD While on Triple Inhaled Therapy
Source: J Clin Med. 2025 Sep 15;14(18):6488. doi: 10.3390/jcm14186488 (PMC12470491; doi:10.3390/jcm14186488)
Supplement: Supplementary file 1 [file jcm-14-06488-s001.zip › jcm-3823053-supplementary.docx]

Article

Clinical Burden of People with Symptomatic and Exacerbating COPD While on Triple Inhaled Therapy ^†^

Anthony De Soyza ^1,^*, Clementine Nordon ^2^, Emily Coak ^3^, Tia Pennant ^3^, Hana Mullerova ^2^, Malin Fageras ^4^,
João Andre Alves ^4^ and Wim Janssens ^5^

^1^ Population and Health Sciences Institute, Faculty of Medical Sciences, Newcastle University, Newcastle NE2 4HH, UK

^2^ Global Medical Evidence, AstraZeneca, Cambridge CB2 0AA, UK

^3^ Adelphi Real World, Bollington SK10 5JB, UK

^4^ Global Medical Affairs, AstraZeneca, 43183 Gothenburg, Sweden

^5^ Department of Respiratory Diseases, University Hospital Leuven, 3000 Leuven, Belgium

***** Correspondence: anthony.de-soyza@newcastle.ac.uk; Tel.: +44-(0)1912227043

^†^ This article is a revised and expanded version of 2 conference papers entitled “Characteristics of exacerbating COPD patients with productive cough and on inhaled triple therapy, by smoking status: a real-world multicountry study” and “Perception of COPD control by physicians managing symptomatic exacerbating patients receiving inhaled triple therapy: a real-world multicountry 2022 survey”, which were presented at the European Respiratory Society Congress, Vienna, Austria, 7–11 September 2024.

# **SUPPLEMENTARY MATERIAL**

# **TABLE S1.** Physician and patient cohorts across countries.

|  |  | Physician survey | Patient record form*  (filled out by the physician) | | Patient self-completion |
| --- | --- | --- | --- | --- | --- |
|  |  | PCPs/respiratory specialists | Consecutive  sample^†^ | Oversample^‡^ | Consecutive  sample |
| **France  (Q3-Q4 2022)** | PCPs | 45 | 295 | 30 | 74 |
|  | Respiratory specialists | 36 | 176 | - | 23 |
| **Germany (****Q4 2022–Q1 2023)** | PCPs | 50 | 294 | 55 | 150 |
|  | Respiratory specialists | 50 | 247 | 5 | 127 |
| **Italy (Q3 2022–Q1 2023)** | PCPs | 51 | 301 | 43 | 64 |
|  | Respiratory specialists | 53 | 259 | - | 71 |
| **Spain  (Q3-Q4 2022)** | PCPs | 52 | 302 | 21 | 125 |
|  | Respiratory specialists | 51 | 240 | - | 86 |
| **UK (Q3 2022–Q1 2023)** | PCPs | 36 | 194 | 18 | 38 |
|  | Respiratory specialists | 40 | 226 | - | 64 |
| **US (Q3 2022–Q1 2023)** | PCPs | 79 | 480 | 19 | 132 |
|  | Respiratory specialists | 58 | 323 | - | 86 |
| **China (Q3-Q4 2022)** | Hospital-based  respiratory specialists | 236 | 844 | - | 835 |
|  |  |  |  |  |  |
| **Total** | | **837** | **4181** | **191** | **1875** |

*Consecutive sample and oversample were collected separately and are mutually exclusive.

^†^Consecutive sample: The next 5-8 patients seen with COPD, per physician.

^‡^Oversample: Completed by physician retrospectively for 1-2 COPD patients prescribed single inhaler triple therapy.

COPD, chronic obstructive pulmonary disease; PCPs, primary care physicians; Q, fiscal quarter.

**TABLE S2.** Additional sociodemographics of patients in the overall, SET+FPC (current/former Smokers, with ≥2 moderate or ≥1 severe Exacerbation in the prior year while receiving Triple inhaled therapy [SET] and frequent productive cough [FPC]), and SET w/o FPC cohorts.

|  | Overall  N=4372 | SET+FPC  n=399 | SET w/o FPC  n=182 | *P* value* |
| --- | --- | --- | --- | --- |
| **Country of inclusion, n (%)** |  |  |  | **<0.0001** |
| France | 501 (11.5) | 55 (13.8) | 14 (7.7) |  |
| Germany | 601 (13.7) | 27 (6.8) | 18 (9.9) |  |
| Italy | 603 (13.8) | 76 (19.0) | 12 (6.6) |  |
| Spain | 563 (12.9) | 95 (23.8) | 36 (19.8) |  |
| UK | 438 (10.0) | 71 (17.8) | 39 (21.4) |  |
| US | 822 (18.8) | 36 (9.0) | 41 (22.5) |  |
| China | 844 (19.3) | 39 (9.8) | 22 (12.1) |  |
| **Employment status, n (%)** |  |  |  | 0.331 |
| Retired | 2395 (54.8) | 285 (71.4) | 120 (65.9) |  |
| Working (full-time/part-time) | 1433 (32.8) | 55 (13.8) | 36 (19.8) |  |
| Unemployed/on long-term sick leave | 252 (5.8) | 41 (10.3) | 18 (9.9) |  |

Notes: The SET+FPC and SET w/o FPC cohorts are subsets of the overall cohort and defined as patients with a history of smoking and ≥2 moderate or ≥1 severe exacerbation while on triple inhaled therapy, with or without FPC, respectively.

**P*-value comparison between the SET+FPC (primary cohort) and SET w/o FPC cohort; statistical analyses were conducted using Chi-Square-tests and Fisher’s Exact tests, as appropriate (*P* <0.05 indicated in bold). No comparisons with the overall cohort were conducted as it includes the SET+FPC and SET w/o FPC cohorts and is only provided for context.

FPC, frequent productive cough; SET, current or former smokers with frequent/severe exacerbations while on triple inhaled therapy; w/o, without.

# **TABLE S3.** Sociodemographics, cardiovascular conditions, COPD-related characteristics, and current treatment duration among patients in the SET+FPC subcohort (current/former Smokers, with ≥2 moderate or ≥1 severe Exacerbation in the prior year while receiving Triple inhaled therapy [SET] and frequent productive cough [FPC]) stratified by smoking status.

|  | SET+FPC,  current smokers  n=150 | SET+FPC,  former smokers  n=249 | *P* value |
| --- | --- | --- | --- |
| **Mean (SD) age, years** | 65.7 (8.6) | 71.2 (9.0) | **<0.0001** |
| **Male, n (%)** | 98 (65.3) | 190 (76.3) | **0.021** |
| **Cardiovascular and metabolic diseases, n (%)** | | |  |
| Hypertension | 87 (58.0) | 169 (67.9) | 0.053 |
| Elevated cholesterol/hyperlipidemia | 37 (24.7) | 77 (30.9) | 0.209 |
| Coronary artery disease | 16 (10.7) | 35 (14.1) | 0.357 |
| Cardiac arrhythmias | 21 (14.0) | 58 (23.3) | **0.027** |
| Congestive heart failure | 6 (4.0) | 31 (12.4) | **0.004** |
| Cerebrovascular disease | 10 (6.7) | 22 (8.8) | 0.569 |
| Angina pectoris | 10 (6.7) | 16 (6.4) | 1.000 |
| Myocardial infarction | 6 (4.0) | 19 (7.6) | 0.201 |
| Diabetes without chronic  complication | 25 (16.7) | 53 (21.3) | 0.298 |
| Diabetes with chronic complication | 8 (5.3) | 16 (6.4) | 0.829 |
| **Asthma codiagnosis, n (%)** | 5 (3.3) | 8 (3.2) | 1.000 |
| **Oxygen therapy use, n (%)** |  |  | 0.694 |
| Ambulatory | 21 (14.0) | 36 (14.5) |  |
| Long-term | 28 (18.7) | 55 (22.1) |  |
| None | 101 (67.3) | 158 (63.5) |  |
| **Most recent BEC (cells/μL)*** |  |  |  |
| Patients with missing data | n=127 | n=215 |  |
| Patients included | n=23 | n=34 |  |
| Mean (SD) | 289.7 (301.5) | 269.3 (213.0) | 0.766 |
| Median (Q1, Q3) | 163.0 (118.0, 320.0) | 215.0 (140.0, 300.0) |  |
| <150, n (%) | 8 (34.8) | 8 (23.5) | 0.400 |
| 150-299, n (%) | 9 (39.1) | 15 (44.1) |  |
| ≥300, n (%) | 6 (26.1) | 11 (32.4) |  |
| **Most recent post-BD FEV_1_  (% predicted)*** |  |  |  |
| Patients with missing data | n=80 | n=132 |  |
| Patients included | n=70 | n=117 |  |
| Mean (SD) | 52.7 (16.1) | 48.8 (16.6) | 0.112 |
| Median (Q1, Q3) | 50.0 (41.0, 63.2) | 48.0 (37.0, 60.5) |  |
| Degree of airflow obstruction (GOLD stage), n (%) |  |  | 0.111 |
| ≥80% (GOLD 1 [mild]) | 7 (10.0) | 6 (5.1) |  |
| 50%-79% (GOLD 2 [moderate]) | 29 (41.4) | 44 (37.6) |  |
| 30%-49% (GOLD 3 [severe]) | 30 (42.9) | 53 (45.3) |  |
| <30% (GOLD 4 [very severe]) | 4 (5.7) | 14 (12.0) |  |
| **Pattern of exacerbations in the prior 12 months** | | |  |
| ≥2 moderate (no severe), n (%) | 43 (28.7) | 85 (34.1) | 0.2575 |
| ≥1 severe (± moderate), n (%) | 107 (71.3) | 164 (65.9) |  |
| Mean (SD) number of exacerbations | 2.5 (1.5) | 2.5 (1.3) | 0.682 |
| **Breathlessness (dyspnea scale), n (%)^†^** | | | 0.927 |
| Grade 0: Only gets breathless after strenuous exercise | 6 (4.0) | 8 (3.2) |  |
| Grade 1: Gets breathless when  hurrying on level ground or walking up a slight incline | 28 (18.7) | 45 (18.1) |  |
| Grade 2: On level ground, walks slower than people of the same age because of breathlessness or has to stop for breath when walking | 50 (33.3) | 92 (36.9) |  |
| Grade 3: Stops for breath after  walking a few minutes on  level ground | 55 (36.7) | 79 (31.7) |  |
| Grade 4: Is too breathless to leave the house or becomes breathless when getting dressed | 11 (7.3) | 25 (10.0) |  |
| Patients with a breathlessness score  grade ≥2 | 116 (77.3) | 196 (78.7) |  |
| **Duration of current treatment, weeks^‡^** | | |  |
| Mean (SD) | 54.6 (67.7) | 55.0 (74.8) | 0.964 |
| Median (Q1, Q3) | 40.0 (12.0, 52.0) | 35.0 (12.5, 55.0) |  |

Notes: The SET+FPC is a subset of the overall cohort and defined as patients with a history of smoking and ≥2 moderate or ≥1 severe exacerbation while on triple inhaled therapy with FPC. Statistical analyses were conducted using T-tests, Fisher’s Exact tests, and Mann-Whitney U tests, as appropriate (*P* <0.05 indicated in bold).

*For patients with available information.

^†^Based on the modified Medical Research Council dyspnea scale.

^‡^Based on physician-reported estimates of treatment duration.

BD, bronchodilator; BEC, blood eosinophil count; COPD, chronic obstructive pulmonary disease; FEV_1_, forced expiratory volume in 1 second; FPC, frequent productive cough; GOLD, Global Initiative for Chronic Obstructive Lung Disease; Q, quartile; SET, current or former smokers with frequent/severe exacerbations while on triple inhaled therapy.

# **TABLE S4.** Patient-reported CAT and EQ-VAS scores for the SET+FPC subcohort (current/former Smokers, with ≥2 moderate or ≥1 severe Exacerbation in the prior year while receiving Triple inhaled therapy [SET] and frequent productive cough [FPC]) stratified by smoking status.

|  | SET+FPC,  current smokers  n=150 | SET+FPC,  former smokers  n=249 | *P* value |
| --- | --- | --- | --- |
| **CAT: total score*** |  |  |  |
| Patients with missing data | n=109 | n=162 |  |
| Patients included | **n=41** | **n=87** |  |
| Mean score (SD) | 24.3 (8.0) | 25.3 (7.4) | 0.471 |
| Median (Q1, Q3) | 25.0 (20.0, 30.0) | 27.0 (22.0, 30.0) |  |
| **CAT cough domain score, n (%)*****^,^**^†^ |  |  |  |
| Patients with missing data | n=109 | n=163 |  |
| Patients included | **n=41** | **n=86** |  |
| <2 | 2 (4.9) | 5 (5.8) | 1.000 |
| ≥2 | 39 (95.1) | 81 (94.2) |  |
| **CAT phlegm/sputum domain score, n (%)*****^,‡^** |  |  |  |
| Patients with missing data | n=109 | n=163 |  |
| Patients included | **n=41** | **n=86** |  |
| <2 | 3 (7.3) | 10 (11.6) | 0.546 |
| ≥2 | 38 (92.7) | 76 (88.4) |  |
| **EQ-VAS*** |  |  |  |
| Patients with missing data | n=109 | n=162 |  |
| Patients included | **n=41** | **n=87** |  |
| Mean (SD) score | 54.6 (20.0) | 52.0 (16.4) | 0.435 |

Notes: The SET+FPC is a subset of the overall cohort and defined as patients with a history of smoking and ≥2 moderate or ≥1 severe exacerbation while on triple inhaled therapy with FPC. Statistical analysis was conducted using T-tests.

*For patients with available information.

^†^The cough item on the CAT is scored on a scale of 0 (“I never cough”) to 5 (“I cough all the time”).

**^‡^**The phlegm/sputum item on the CAT is scored on a scale of 0 (“I have no phlegm in my chest at all”) to 5 (“My chest is completely full of phlegm”).

CAT, COPD Assessment Test; COPD, chronic obstructive pulmonary disease; EQ-VAS, European Quality of Life Visual Analogue Scale; FPC, frequent productive cough; Q, quartile; SET, current or former smokers with frequent/severe exacerbations while on triple inhaled therapy.

# **FIGURE S1.** Respiratory symptoms frequently reported by physicians for patients in the SET+FPC subcohort (current/former Smokers, with ≥2 moderate or ≥1 severe Exacerbation in the prior year while receiving Triple inhaled therapy [SET] and frequent productive cough [FPC]) stratified by smoking status.


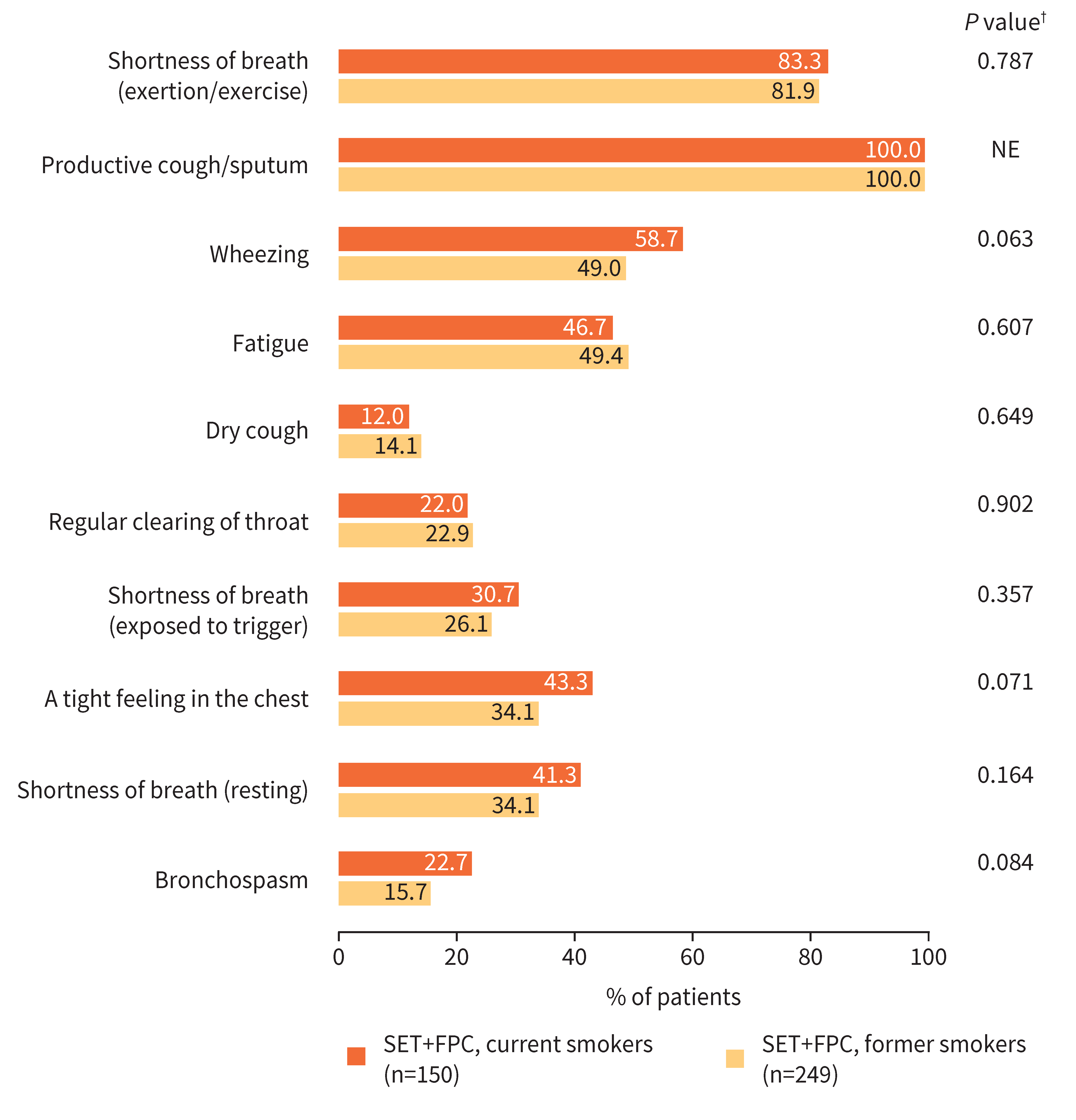


Notes: The SET+FPC is a subset of the overall cohort and defined as patients with a history of smoking and ≥2 moderate or ≥1 severe exacerbation while on triple inhaled therapy with FPC.

^†^Statistical analysis was conducted using Fisher’s Exact tests.

FPC, frequent productive cough; NE, not evaluable; SET, current or former smokers with frequent/severe exacerbations while on triple inhaled therapy.

# **FIGURE S2.** Physician satisfaction with COPD control for the SET+FPC subcohort (current/former Smokers, with ≥2 moderate or ≥1 severe Exacerbation in the prior year while receiving Triple inhaled therapy [SET] and frequent productive cough [FPC]) stratified by smoking status.


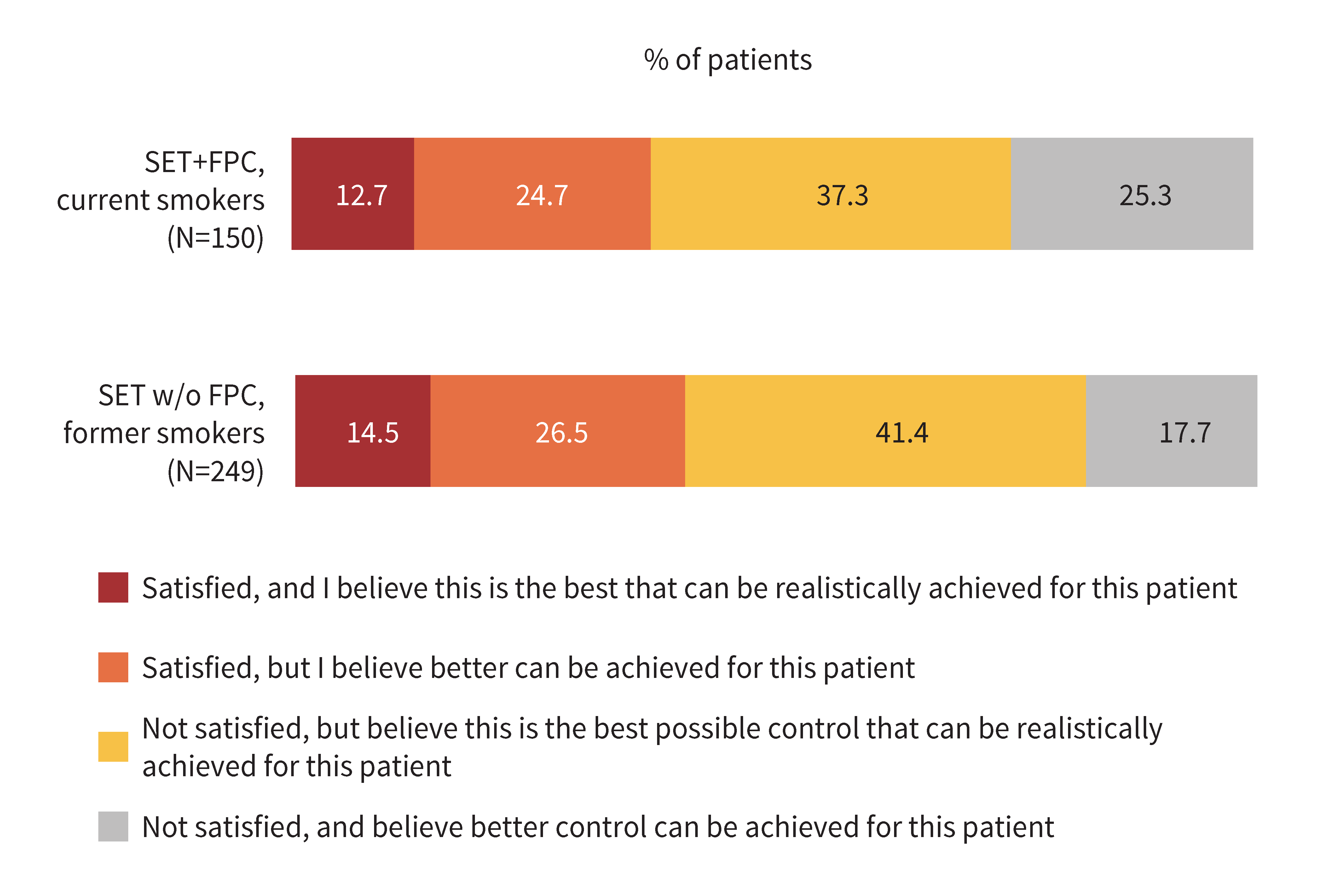


Notes: The SET+FPC is a subset of the overall cohort and defined as patients with a history of smoking and ≥2 moderate or ≥1 severe exacerbation while on triple inhaled therapy with FPC. Statistical analysis was conducted using the Mann-Whitney U test; *P*=0.175.

COPD, chronic obstructive pulmonary disease; FPC, frequent productive cough; SET, current or former smokers with frequent/severe exacerbations while on triple inhaled therapy.

# **FIGURE S3.** Physician-reported severity of COPD in patients, from time of current treatment initiation to time of survey for the overall cohort, SET+FPC (current/former Smokers, with ≥2 moderate or ≥1 severe Exacerbation in the prior year while receiving Triple inhaled therapy [SET] and frequent productive cough [FPC]) cohorts (overall and stratified by smoking status), and SET w/o FPC cohort.


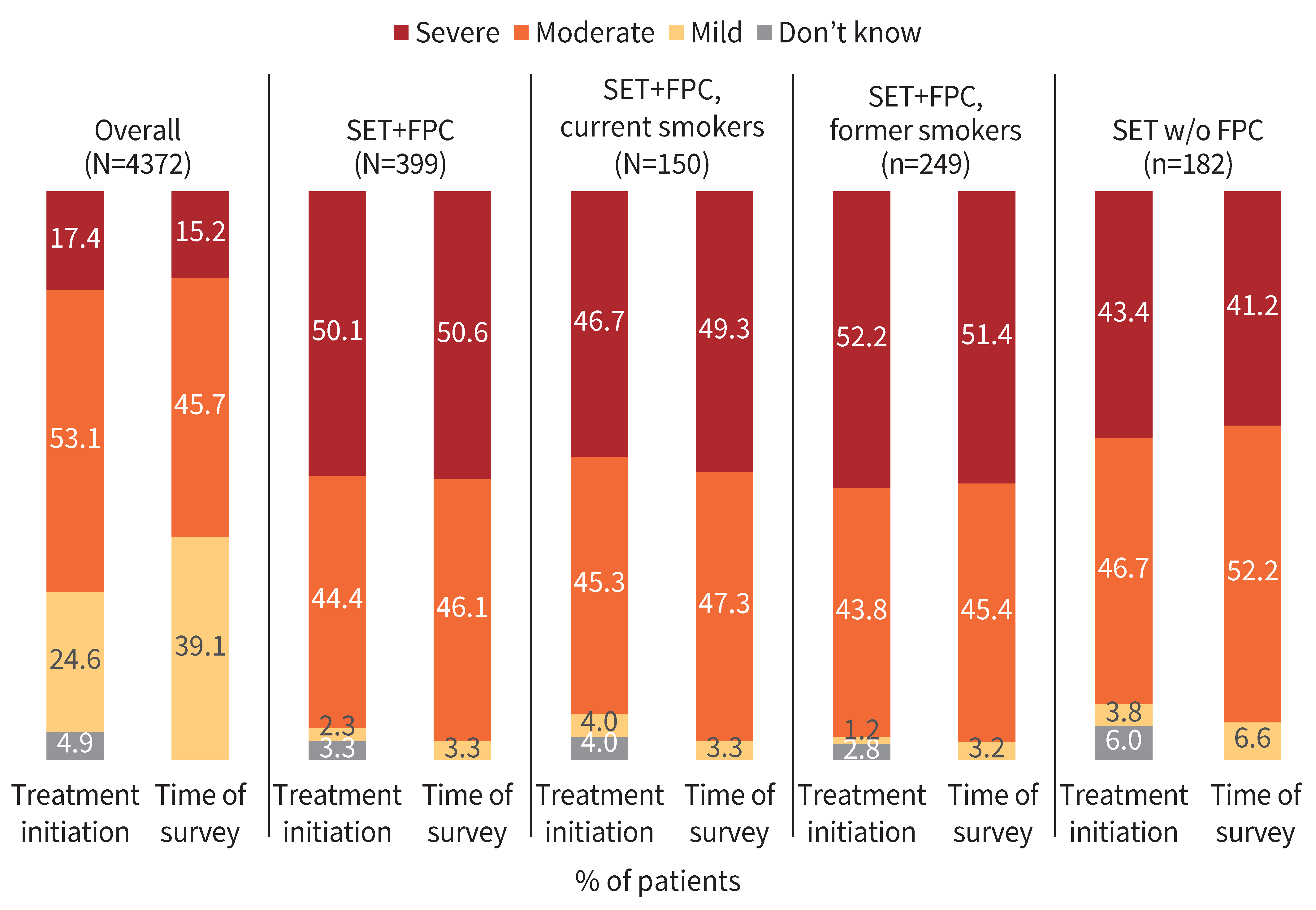


Notes: The SET+FPC and SET w/o FPC subcohorts are subsets of the overall cohort and defined as patients with a history of smoking and ≥2 moderate or ≥1 severe exacerbation while on triple inhaled therapy, with or without FPC, respectively.

COPD, chronic obstructive pulmonary disease; FPC, frequent productive cough; SET, current or former smokers with frequent/severe exacerbations while on triple inhaled therapy; w/o, without.
